# Cognitive biases and moral characteristics of healthcare workers and their treatment approach for persons with advanced dementia in acute care settings

**DOI:** 10.3389/fmed.2023.1145142

**Published:** 2023-06-22

**Authors:** Meira Erel, Esther-Lee Marcus, Freda DeKeyser Ganz

**Affiliations:** ^1^Henrietta Szold Hadassah-Hebrew University School of Nursing, Jerusalem, Israel; ^2^Division of Geriatrics, Herzog Medical Center, Jerusalem, Israel; ^3^Faculty of Health and Life Sciences, Jerusalem College of Technology, Jerusalem, Israel

**Keywords:** acute care settings, cognitive bias, dementia, moral courage, moral sensitivity, palliative care

## Abstract

**Introduction:**

Palliative care (PC) delivery for persons with advanced dementia (AD) remains low, particularly in acute-care settings. Studies have shown that cognitive biases and moral characteristics can influence patient care through their effect on the thinking patterns of healthcare workers (HCWs). This study aimed to determine whether cognitive biases, including representativeness, availability, and anchoring, are associated with treatment approaches, ranging from palliative to aggressive care in acute medical situations, for persons with AD.

**Methods:**

Three hundred fifteen HCWs participated in this study: 159 physicians and 156 nurses from medical and surgical wards in two hospitals. The following questionnaires were administered: a socio-demographic questionnaire; the Moral Sensitivity Questionnaire; the Professional Moral Courage Scale; a case scenario of a person with AD presenting with pneumonia, with six possible interventions ranging from PC to aggressive care (referring to life-prolonging interventions), each given a score from (−1) (palliative) to 3 (aggressive), the sum of which is the “Treatment Approach Score;” and 12 items assessing perceptions regarding PC for dementia. Those items, the moral scores, and professional orientation (medical/surgical) were classified into the three cognitive biases.

**Results:**

The following aspects of cognitive biases were associated with the Treatment Approach Score: representativeness—agreement with the definition of dementia as a terminal disease and appropriateness of PC for dementia; availability—perceived organizational support for PC decisions, apprehension regarding response to PC decisions by seniors or family, and apprehension regarding a lawsuit following PC; and anchoring—perceived PC appropriateness by colleagues, comfort with end-of-life conversations, guilt feelings following the death of a patient, stress, and avoidance accompanying care. No association was found between moral characteristics and the treatment approach. In a multivariate analysis, the predictors of the care approach were: guilt feelings about the death of a patient, apprehension regarding senior-level response, and PC appropriateness for dementia.

**Conclusion:**

Cognitive biases were associated with the care decisions for persons with AD in acute medical conditions. These findings provide insight into the potential effects of cognitive biases on clinical decisions, which may explain the disparity between treatment guidelines and the deficiency in the implementation of palliation for this population.

## 1. Introduction

The recognition of dementia as a progressive terminal disease, where palliative care (PC) is appropriate for the late advanced stage, is rapidly increasing. However, PC delivery for persons with advanced dementia (AD) remains low (1, 2). The treatment approach for persons with AD presenting with acute or life-threatening medical situations can be viewed on a spectrum, ranging from aggressive care to PC. However, this treatment approach is a function of the goals of care, where aggressive care is broadly defined as burdensome interventions and therapeutic regimens designed to preserve and prolong life (“life-prolonging care”), whereas PC is focused on improving quality of life, maximizing comfort, and preventing suffering. Life-prolonging care focuses on normalizing the medical state in the face of acute illness as a form of curative proactive approach. This approach eliminates medical crises rather than simply minimizing their impact (3).

Studies have investigated factors associated with this low level of PC delivery, including demographic and work-related characteristics of healthcare workers (HCWs), such as administrative regulations (financial reimbursement, access to PC, time constraints, staff shortage, and services integration and collaboration, education, knowledge, or communication skills) (4, 5), and professional orientation (surgical vs. medical) (6). However, another potentially important component in deciding whether to provide PC is the process of clinical thinking or reasoning by the HCW when making a treatment decision, which is considered the core of healthcare practice (7). The decision-making process can involve two types of thinking processes, which include fast and intuitive or slow and full of effort (8). The fast, intuitive thinking processes may lead to cognitive biases, which refer to how decisions are made based on thinking patterns and judgments that deviate from a rational thinking process (8, 9).

Clinical thinking and reasoning, particularly those involving decisions that set the sensitivity course for or against life-sustaining treatment choices, also involve moral aspects, including moral courage and sensitivity. Moral courage is “a prosocial behavior” intended to enforce ethical norms, without considering one’s cost (10). In our context, moral courage refers to the decision to act differently from patterns of traditionally accepted treatment in acute care settings that focuses on saving a life. In clinical medicine, moral courage can be inhibited by organizational constraints, hierarchical structures, or career concerns (10, 11). Furthermore, it has been reported that higher moral courage levels among HCWs increase the quality of care and patient safety (12). The HCWs’ gender (males choose more assertive behavior and present a higher ability to influence patient care), individual characteristics, inner motivation, and self-confidence were also associated with acting courageously (12–14).

A second moral aspect is moral sensitivity, which is defined as the sense that bridges the gap between moral knowledge and actual behavior (15). Many studies have investigated moral sensitivity in the context of HCWs. They found that the level of moral sensitivity among HCWs ranged from low to moderate (16–18), except for one recent study conducted during the coronavirus disease 2019 pandemic, which found a high level of moral sensitivity (19). Similar to moral courage, moral sensitivity was found to relate positively to patient safety and the quality of care, and the two concepts were reported to relate to one another (18, 19). However, to the best of our knowledge, the association between these moral aspects and clinical thinking and reasoning has not been reported in the literature.

Faulty clinical thinking or reasoning may cause severe adverse effects on patients (20). If the HCW’s reasoning is not sound, an incorrect clinical decision could be made, leading to medical complications. The prevailing assumption is that clinical reasoning is a rational, objective process (21, 22). However, treatment decisions are made within a complex organizational framework, which can stimulate cognitive biases that may lead to errors or irrational decisions. Additionally, clinical decisions can be based on common misinterpretations of clinical information or irrelevant factors that can produce significant diversity in decisions and judgments (8, 23, 24). Whether inherent or learned, cognitive processes may deviate from the rules of logic and probability (20). Furthermore, work environment-related factors may also unconsciously influence judgment and clinical thinking (22, 25–27). These factors may be sublimated in the hospital acute care settings, where the stressful environment may enhance the need to make fast decisions based on previous clinical experience and personal judgment (26–29).

Therefore, this study hypothesized that the cognitive biases, representativeness, availability, and anchoring, might explain the gap between numerous evidence-based recommendations to provide PC to persons with AD and actual practice in acute settings (30, 31). Representative bias (sometimes referred to as framing bias) is the tendency to judge a situation according to its visibility, use categorical schematic thinking, and rely on clues and objective evidence that matches known patterns (8). However, this type of bias may occur when information is presented or observed in a certain manner that emphasizes and highlights one aspect of the situation, thereby subconsciously leading to intuitive conclusions. When representative bias occurs, judgment is prejudiced by the likelihood of a common or ‘representative’ judgment that matches only a part of the clinical data. Simultaneously, other known information is ignored, and missing information is not explored, leading to a considerably narrow view of the clinical picture (26).

However, moral sensitivity may mitigate the effect of this biased judgment. For example, in our context, the current common or representative judgment is to continue aggressive, potentially burdensome, acute care for all patients admitted to acute care hospitals, including those with AD at the end of life. HCWs with a high level of moral sensitivity might realize the ethical dilemma in this approach, which many consider as futile care. Additionally, this healthcare provider would consider other aspects of medical treatment, including the patient’s and caregiver’s values and preferences. Therefore, a low level of moral sensitivity might be associated with a higher likelihood of using representative bias.

Availability bias is defined as placing greater value on information that comes to mind quickly, tending to offer increased credibility to those thoughts that arise easily (25). Additionally, due to emotional arousal, vivid thoughts are frequently easily recalled and imagined, making them appear more important (32). For example, previously, a physician suggested PC to the family member of a person with AD. Unfortunately, the family member reacted negatively, causing the physician to feel guilty and uncomfortable. These negative reactions tend to prevent the physician from suggesting PC when a similar situation arises.

However, if this same physician had a high level of moral courage, they would be more likely to make a comparable suggestion in similar situations, despite the fear of possible negative consequences. Therefore, a low level of moral courage might be associated with a higher likelihood of influence of the availability bias. Therefore, we hypothesized that HCWs with high moral sensitivity or courage would be more likely to consider PC as a treatment approach.

Anchoring bias is the tendency to base decisions and judgments on a recognizable anchor point perceived as a comfortable zone (33). This bias may lead to passive thinking, causing the clinician to think about only accepted/default care options (valued as the acceptable care norm in a certain setting and work environment) without considering other care alternatives, thereby effectively preventing hesitation, uncertainty, and a sense of insecurity (8, 22). The anchor point is acquired during professional and organizational training and socialization (34).

Research about cognitive biases in the context of medical care is a growing field (35). However, only limited studies focusing on medical personnel, particularly those caring for persons with AD, are available (23). The empirical literature on cognitive biases’ potential effect on moral decisions is also limited (36), with unsatisfactory attention regarding the potential distortion of bioethics medical work (37). A few studies assessing cognitive biases that refer to older adults included symptom management and heuristics (38–40), whereas other studies focused on the influence of cognitive biases on medical diagnosis in the general patient population (33, 41, 42). However, examination of the impact of cognitive biases on the clinical decisions that result in diagnostic inaccuracies, medical errors, and patient outcomes is lacking in research, and existing clinical guidelines and recommendations have not yet been used as a framework or measure for determining care decision errors (24). This study used clinical guidelines from recognized and accepted geriatric organizations to outline an appropriate treatment approach for persons with AD at the end of life (43–45).

Considering the insufficient PC in acute settings for persons with AD (46–48), this study aimed to examine the cognitive reasoning processes of HCWs, by determining the association between cognitive biases, assessed using care decisions-related items for persons with AD, including moral sensitivity and courage, and the treatment approach for those with AD presenting with an acute potentially life-threatening medical situation. The study hypothesized that a correlation exists between these biases and the care approach. We performed a secondary analysis of data retrieved from the qualitative part of a mixed-methods study assessing the decision-making of HCWs regarding acute medical situations in persons with AD (31, 49). To the best of our knowledge, the effect of cognitive biases on the treatment approach in this setting has not been reported in the literature.

## 2. Materials and methods

This study was part of the quantitative analysis of a mixed-methods study (quantitative/qualitative) investigating factors associated with treatment approaches for persons with AD in emergency medical situations in acute care hospitals.

### 2.1. Participants and settings

A convenience sample of physicians and nurses working in medical and surgical departments at two tertiary university-affiliated hospitals in Israel was used. The exclusion criterion included having any postgraduate formal geriatric and/or PC training. This criterion was selected since such training could bias the results by influencing attitudes and knowledge of PC. Among the 320 participants, only 5 reported this training and were excluded from the final sample. Participants were recruited during department staff meetings.

### 2.2. Questionnaires

The following five questionnaires were used in this study. (1) A socio-demographic questionnaire, including age, gender, work experience (years), profession (nurse/physician), position (senior/junior), and ward (medical vs. surgical). (2) The Moral Sensitivity Questionnaire – MSQ (50). This questionnaire was designed to measure the level of moral sensitivity. The questionnaire included 27 items on a 1–7 Likert scale, where a higher score reflected a higher level of moral sensitivity. (3) The Professional Moral Courage Scale – PMCS (51). This questionnaire was designed to measure the level of moral courage. It included 15 questions on a 1–7 Likert scale, where higher scores reflected a higher level of moral courage. The final score in both scales was calculated by the sum of the items’ scores divided by the number of items. A score of 1–2 signified a low level of moral sensitivity/moral courage, whereas scores of 3–5 and 6–7 indicated moderate and high levels, respectively. Both scales were forward- and back-translated into Hebrew according to the Brislin (52) method. Alpha Cronbach values of the MSQ and the PMCS in this study were 0.87 and 0.93, respectively, similar to the previous reports among nurses in the literature (53–56) (see Supplemental Files 1a and 1b for the Hebrew version of the MSQ and PMCS tools, respectively). (4) The authors designed the fourth questionnaire to evaluate the treatment approach for persons with AD in emergency life-threatening situations (6, 31). The treatment approach was measured by analyzing responses to a hypothetical scenario of a person with AD presenting with aspiration pneumonia and acute respiratory failure in an acute care hospital. The patient’s description in the clinical scenario was based on parameters from a validated scale [Functional Assessment Staging Test (FAST)] and Hospice guidelines for estimating <6 months survival in a patient with dementia indicating an advanced state of dementia with limited life expectancy (FAST stage 7c and all features of 6A through 7c and at least one of the following: aspiration pneumonia, pyelonephritis or upper urinary tract infection, septicemia, stages 3 or 4 pressure ulcer multiple, recurrent fever after treatment with antibiotics, and eating problems) (43). Participants were asked to indicate which medical interventions or treatments they would choose from a list of six possible interventions, ranging from intubation and mechanical ventilation (reflecting an aggressive treatment approach) to analgesia or sedation (signifying a PC treatment approach). Other possible interventions included were laboratory tests, intravenous fluid infusion, and antimicrobial therapy. Furthermore, each medical treatment choice was given a score ranging from −1 to 3, and more invasive/burdensome choices received higher scores. Palliative measures received a score of −1, whereas not choosing them was scored +1. The sum of the scores of the chosen interventions was termed the “Treatment Approach Score” and ranged from −1 to 11 (see Supplemental file 2a for the Questionnaire and Supplemental file 2b for the Hebrew Version of this Questionnaire). The authors designed this questionnaire and reviewed it for content validity using a panel of six geriatricians working in acute care settings (6). The fifth questionnaire included 12 items that assess potential components related to the following three cognitive biases: representativeness, availability, and anchoring. This questionnaire is part of a larger questionnaire that assessed practices and perceptions regarding PC for persons with AD (6, 31). Participants were asked to rate their level of agreement with the various statements on a 5-point Likert scale. A higher score represented a more aggressive care approach. The included items that were selected from the questionnaire (mentioned above), the moral scores (sensitivity and courage), and professional orientation (medical/surgical) were classified into the three biases according to their content after a literature review of the three cognitive biases and their possible association with clinical decision-making regarding persons with AD (30). This procedure was performed by consensus of all authors. The items for each type of bias are as follows:

#### 2.2.1. Representativeness bias

This bias included three items that have an expected impact on the visibility of the person with AD (as described in the scenario) considered to be in a terminal state by the provider depending on the degree of knowledge of the course of dementia and its stages and the sensitivity in identifying an end-of-life situation. These items are as follows: (1) the perception of dementia as a terminal disease that threatens life; (2) the frequency of assessment of the stage of dementia by the provider; (3) the perception of whether PC is appropriate for persons with AD. Additionally, the level of moral sensitivity (measured using the MSQ questionnaire) that may promote the identification of an ethical dilemma related to an end-of-life state was included in the analysis of the representativeness bias.

#### 2.2.2. Availability bias

Four items were included under this bias indicating concerns, when considering PC, that may arise and influence the choice of this approach for a person with AD in the terminal stage. These items are as follows: (1) lack of organizational support for PC decisions, (2) apprehension regarding senior-level response to PC decisions, (3) apprehension regarding a lawsuit following PC decisions, and (4) fear of family response to PC decisions. The level of moral courage (measured using the PMCS questionnaire), which can moderate these concerns, was included in the analysis of the availability bias.

#### 2.2.3. Anchoring bias

The five items under this bias aimed to examine the care norm (according to the provider’s perception of the mindset of his colleagues) and factors that may lead to reluctance in choosing palliation and hence a preference for the care that is perceived as familiar and safe. These items are as follows: (1) colleagues’ perception of PC as appropriate for persons with AD; (2) feeling comfortable with conducting end-of-life conversations; (3) guilt felt about the death of a person with AD, (4) ability to make medical care decisions for persons with AD, and (5) care of a person with AD causing stress and avoidance. Additionally, the professional orientation, medical vs. surgical, which may influence the norms of care, was included in the anchoring bias.

Using reliability statistics, Cronbach’s Alpha on the 12 standardized items included in this analysis reached a value of 0.66.

### 2.3. Ethical approval

Ethical approval was received from the ethics committee of each hospital (case number 5535-18-SMC; 0027-19-HMO). In addition, the health professionals received oral and written information about the study, and written consent was obtained.

### 2.4. Statistical methods

Descriptive statistics were used to report on study participants’ characteristics and setting variables (ward, profession, position, and work experience). Dichotomous and categorical variables are expressed as percentages and continuous variables are presented as mean, standard deviation, median, and range.

Univariate and multivariate generalized linear models were performed to examine the association between the items relating to representativeness, availability, and anchoring biases and the treatment approach (Treatment Approach Score). The items included in the various cognitive biases were treated as continuous, ordinal variables on a 5-scale, reflecting the level of agreement with various statements. In contrast, professional orientation was entered into the statistical models as a categorical variable (medical vs. surgical). Moral courage and sensitivity indices were treated as continuous variables ranging from a score of 1–7. A backward elimination method was applied by removing variables that did not reach statistical significance in the regression models after adjusting for other covariates. The adjusted covariates were work experience (years), profession (physician/nurse), and position (senior/junior). Separate univariate models were run for each type of bias, and a joint multivariate model, including all biases items, was applied to assess factors associated with the treatment approach. Furthermore, the beta coefficients and their respective standard errors (SEs) were derived from the regression models, representing the degree of change in the outcome variable (Treatment Approach Score) for every 1 unit of change in the predictor variable. A positive beta coefficient indicated that for every 1-unit increase in the predictor variable (increment of 1 unit in the Likert scale), the outcome variable (Treatment Approach Score) would increase by the beta coefficient value. If the beta coefficient was negative, the interpretation is that for every 1-unit increase in the predictor variable, the outcome variable would decrease by the beta coefficient value. All statistical analyses were conducted using the SPSS version 27, and statistical significance was set at *p* < 0.05, using two-tailed tests.

## 3. Results

Overall, 315 HCWs completed the questionnaire. The sample was almost equally divided between the professions (nurses/physicians) and gender. Approximately 40 and 30% of the participants were from surgical wards and senior positions, respectively (Table 1).

**Table 1:** Socio-demographic characteristics of the participants (*N* = 315).

Age (years)	Mean ± SD	35.5 ± 9.4
	Median	33.0
	Range	24–71
Work experience (years)	Mean ± SD	7.7 ± 8.6
	Median	4.0
	Range	1–50
Gender	Male, *n* (%)	148 (47.0)
	Female, *n* (%)	167 (53.0)
Profession	Physician, *n* (%)	159 (50.4)
	Nurse, *n* (%)	156 (49.6)
Position	Senior, *n* (%)	97 (30.8)
	Junior, *n* (%)	218 (69.2)
Ward	Medical, *n* (%)	190 (60.3)
	Surgical, *n* (%)	125 (39.7)

SD, Standard deviation.

### 3.1. The level of moral sensitivity and moral courage

The mean score of the MSQ was 5.0 ± 0.7, and 10.9, 82.8, and 6.3% reported low, moderate, and high levels of moral sensitivity, respectively. The mean score of the PMCS was 5.0 ± 0.9, and 6.4, 62.8, and 30.8% reported low, moderate, and high levels of moral courage, respectively.

### 3.2. The distribution of the answers to the items

The distribution of the answers to the items included in this analysis was previously reported by the authors (6, 31). Briefly, 48.6% of the participants “strongly agreed”/“agreed” with the statement that dementia is a terminal progressive disease that eventually threatens life; 32.4% never or rarely performed assessment of the stage of dementia; 90.4% had positive (“always/very often/often”) attitude toward PC being appropriate for persons with dementia; 27.1% “strongly agreed”/“agreed” with the statement that there is “Lack of organizational support for PC decisions for persons with dementia;” 33.8% reported on a “very high”/“high” level of apprehension regarding senior-level response to PC decisions; 33.1% reported a “very high”/“high” level of apprehension regarding lawsuit following PC decisions; 69.7% had a “very high”/“high” fear of family response to PC decisions; 73.6% perceived PC appropriate (“always”/“very often”/“often”) for persons with dementia by colleagues; 58.0% felt comfortable (“always”/“very often”/“often”) with conducting end-of-life conversations with family members; 59.9% “strongly disagreed”/“disagreed” with the statement that “I perceive a death of a patient with dementia as a failure accompanied with guilt; 56.7% reported having ability (“strongly agree”/“agree”) to make medical care decisions for persons with dementia; and 24.5% “strongly agreed”/“agreed” that caring for persons with AD make them feel stressed and avoidant.

### 3.3. Representativeness bias

Three items and the moral sensitivity score were related to representativeness bias (see Table 2). A higher level of agreement with the items “dementia is a terminal disease” and “PC is appropriate for persons with dementia” were associated with a higher preference toward a PC approach in the univariate analysis. However, no association was observed between performing a disease stage evaluation or the level of moral sensitivity and the treatment approach. In a multivariate analysis of all representativeness items and the level of moral sensitivity [adjusted for work experience (years), profession (physician/nurse), and position (senior/junior)], only the appropriateness of PC for dementia was statistically significantly associated with the treatment approach. Participants who reported higher levels of disagreement with the statement “PC is appropriate for persons with dementia” reported a higher mean treatment approach score of 0.72 for every increase in 1 point of the Likert score of this item, representing a less PC approach (*B* = 0.72, SE = 0.20; *p* < 0.001) (Table 2).

**Table 2:** Univariate and multivariate analyses of the association between representativeness, availability, and anchoring biases and the treatment approach score.

Type of bias	Univariate model				Multivariate model*		
	Variable	*B*	*SE*	*P*	*B*	*SE*	*P*
Representativeness bias	Dementia is a terminal disease^1^	0.40	0.14	0.006	0.26	0.15	0.08
	Performing a disease stage evaluation^2^	0.17	0.14	0.2	−0.05	0.15	0.76
	PC is appropriate for dementia^2^	0.81	0.18	<0.001	0.72	0.20	<0.001
	Moral sensitivity (MSQ)	−0.29	0.20	0.15	−0.19	0.20	0.35
Availability bias	Lack of organizational support for PC decisions for persons with AD^3^	0.32	0.16	0.04	−0.15	0.17	0.41
	Apprehension regarding senior-level response to PC decisions^3^	0.59	0.15	<0.001	0.88	0.18	<0.001
	Apprehension regarding a lawsuit following PC decisions^3^	0.92	0.14	<0.001	0.13	0.18	0.48
	Fear of family response to PC decisions^3^	0.48	0.18	0.008	0.17	0.18	0.37
	Moral courage (PMCS)	−0.09	0.17	0.60	−0.10	0.16	0.53
Anchoring bias	Perceived PC appropriate for persons with AD by colleagues^1^	0.70	0.19	<0.001	0.60	0.18	0.001
	Comfortable with conducting end-of-life conversations^1^	0.65	0.14	<0.001	0.60	0.17	<0.001
	The guilt felt about the death of a person with AD^4^	0.73	0.16	<0.001	0.61	0.16	<0.001
	Ability to make care decisions for the person with AD^1^	0.23	0.15	0.13	−0.30	0.18	0.09
	Care of persons with AD causes stress and avoidance^4^	0.32	0.16	0.04	0.07	0.16	0.68
	Professional orientation (medical vs. surgical)	−0.09	0.33	0.80	0.33	0.32	0.30

AD, advanced dementia; MSQ, moral sensitivity questionnaire; PC, palliative care; PMCS, professional moral courage scale.

* Adjusted for work experience (years), profession (physician/nurse), and professional position (senior/junior).

1 Level of agreement from 1 (totally agree) to 5 (totally disagree).

2 Frequency from 1 (always) to 5 (never).

3 Level of apprehension regarding delivering PC from 1 (very low) to 5 (very high).

4 Level of agreement from 1 (totally disagree) to 5 (totally agree).

### 3.4. Availability bias

In the univariate analysis, the four items, including the apprehension regarding senior-level response to PC decisions, apprehension regarding a lawsuit following PC, lack of organizational support for PC decisions for persons with AD, and fear of a negative reaction to PC decisions from family members, were statistically significantly associated with the treatment approach (Table 2). However, the level of moral courage was not associated with the treatment approach. In the multivariate analysis of all items related to the availability bias, controlling for work experience, profession, and position (senior/junior), apprehension regarding senior responses to PC decisions remained an independent factor statistically significantly associated with the treatment approach score (*B* = 0.88, SE = 0.18; *p* < 0.001) (Table 2).

### 3.5. Anchoring bias

Four of the five anchoring bias items were statistically significantly associated with the treatment approach in the univariate analyses. These items were the perception by colleagues that PC is appropriate for persons with AD, being comfortable with conducting end-of-life conversations, guilty feelings about the death of a person with AD, and care of those with AD causing stress and avoidance. In the multivariate analysis, three of these items remained statistically significantly associated with treatment scores (Table 2). A mean increase of 0.60 in the treatment approach score (representing a less PC approach) was found for every point increase in the Likert score when participants disagreed with the statements “Perceived PC is appropriate for persons with AD by colleagues” (*B* = 0.60, SE = 0.18; *p* = 0.001), reported being more uncomfortable with conducting end-of-life conversations (*B* = 0.60, SE = 0. 17; *p* < 0.001), or feeling guilt over the death of a person with AD (*B* = 0.61, SE = 0.16; *p* < 0.001). However, the ability to make care decisions for persons with AD and professional orientation were not associated with the treatment approach.

### 3.6. Predictors of treatment approach

Table 3 summarizes the variables that were significantly and independently associated with the Treatment Approach Score in the multivariate model. Three items representing the three types of biases remained statistically significant in the multivariate model. The agreement that PC is appropriate for dementia was associated with lower treatment scores, representing the choice of a more PC approach (*B* = 0.50, SE = 0.18; *p* = 0.005). Participants who agreed that feeling guilty about the death of a person with AD or being apprehensive about a senior-level response to PC decisions were less likely to choose a PC approach. The mean treatment approach score increased by 0.39 and 0.58, respectively, for every point increase in the Likert score of those items.

**Table 3:** Joint model including all biases associated with the treatment approach score.

Type of bias		*B*	*SE*	*p*
Representativeness bias	PC is appropriate for dementia^1^	0.50	0.18	0.005
Availability bias	Apprehension regarding senior-level response to PC decisions^2^	0.58	0.16	<0.001
Anchoring bias	Comfortable with conducting end-of-life conversations^3^	0.26	0.14	0.080
Anchoring bias	The guilt felt about the death of the person with AD^4^	0.39	0.16	0.012
Anchoring bias	Perceived PC appropriate for persons with AD by colleagues^3^	0.32	0.18	0.076

AD, advanced dementia; PC, palliative care.

1 Frequency from 1 (always) to 5 (never).

2 Level of apprehension delivering PC from 1 (very low) to 5 (very high).

3 Level of agreement from 1 (totally agree) to 5 (totally disagree).

4 Level of agreement from 1 (totally disagree) to 5 (totally agree).

## 4. Discussion

Our findings confirm that several items related to the three biases studied, including representativeness, availability, and anchoring, were associated with the treatment approach to acute medical life-threatening situations for persons with AD. Additionally, in the multivariate analysis, at least one item of each bias was associated with the treatment approach.

We hypothesized that items related to representativeness bias, including moral sensitivity, would be associated with treatment choices for persons with AD in an acute medical situation. Limited recognition of dementia as a terminal disease and the lack of perception of PC as adequate for persons in its advanced stage were related to aggressive treatment choice in the univariate analysis. It appears that these factors may bias clinical thinking due to their influence on the representativeness of the patient in the eyes of the HCW. Consistent with this study’s findings, HCWs’ negative perceptions of PC for persons with dementia have been reported as a barrier to PC and were associated with continued delivery of aggressive care (49, 57–60). Given that PC is not considered appropriate for application to a person with AD by some of the participants in this study suggests that the visibility and representation of the person as being at the end of life are not accepted, and the judgment of the clinical situation is diverted in favor of a narrow clinical consideration focused on the present.

Therefore, we hypothesized that identifying the stage of dementia may promote the visibility of persons with AD as being at the end of their lives and their suitability for considering PC. However, no association was found between stage evaluation and treatment approach. Nevertheless, others claimed that including an assessment of the illness trajectory by assessing the stage of dementia in decision-making is required for goals of care decisions and can lead to changing the goals from curative to PC (61, 62).

Moral sensitivity scores in our study were relatively homogeneous, concentrating on the moderate level, with a minority of participants reporting very high or low levels. Previous studies that examined professional moral sensitivity using a similar scale among HCWs found a low-moderate level, despite exposure to ethically sensitive events as part of their profession (17, 18, 63). This study hypothesized that the high moral sensitivity level of HCWs would shape and bias the thinking to identify the person’s end-of-life situation. Therefore, participants who report a high level of moral sensitivity will choose the treatment options to alleviate suffering. Nevertheless, no association was found between moral sensitivity and the treatment approach. This finding may be explained by the low impact of moral sensitivity on professional practice. Indeed, medical education is mainly based on algorithms and evidence-based medicine and may lead to mechanical or limited thinking and exclude aspects of moral judgment, such as moral sensitivity (25, 64). It is similar to judicial decisions that evaluate medical negligence based on the criteria of “the reasonable HCW.” This means that the law accepts that HCWs’ behavior should be judged by how other professionals behave and not necessarily by ideal moral standards (65).

This study’s results indicate that respondents may lack an understanding of the nature of dementia and the suitability of PC for these persons. This study’s participants were acute care hospital HCWs, who most probably focus on clinical data related to acute medical conditions and are less concerned with progressive chronic conditions. Additionally, this may create an intuitive way of thinking expressed here as representativeness bias. Confirming a bias is performed either by observing that a factor, which should not affect judgment, has a statistical effect on it or a factor that should influence the judgment does not (25). This thinking pattern may ignore information, indicating other possible treatment options, thereby raising the risk of delivering more aggressive care. Different studies have reported the involvement of representativeness bias, also known as “framing bias” or “halo bias”’ in the medical context. These studies reported the impact of the general impression that dominated situation judgment (36). For example, a study that examined the effect of how a hypothetical clinical trial comparing an old and new drug was presented to clinicians found that its presentation of the benefit regarding relative mortality reduction led to a more favorable opinion about the new drug compared with the presentation of the benefit in terms of absolute morality or survival (66).

We hypothesized that the availability bias, including moral courage, is associated with the treatment approach administered to persons with AD. Indeed, we found a statistically significant association between increased concerns regarding organization support, senior-lever response, lawsuit, and family response when providing PC and the chosen treatment approach. This correlation may be because of how these concerns potentially bias the thinking process, consciously or unconsciously. Previous studies have shown that fear of personal sanctions may arise when providing PC. For example, in a systematic review, Paque et al. (67) found that perceived lack of organizational support and apprehension from senior-level HCWs acted as barriers to de-prescribing medications for persons with a life-limiting illness. The hierarchical nature of health organizations and a sense of privilege and status can cause those at the top to treat others below them with disrespect (68). Additionally, fear of senior staff was described by trainee doctors, who experienced intimidation that impacted their decision-making (69, 70). Others also found that legal concerns, fear of malpractice claims, and apprehension associated with family response to PC delivery predicted more aggressive care for people with dementia (71–73). Such apprehensions also raised the likelihood of aggressive care in end-of-life among intensive care HCWs, despite being understood by the provider as futile (74). Another barrier to PC delivery is limited knowledge and understanding of the law, which may result in less appropriate care for the person at the end of life (75–78).

However, our findings did not support the hypothesis that moral courage is associated with the treatment approach. The study assumed that high moral courage would moderate the bias of the various concerns that may accompany the decision to provide PC in their conscious existence, thereby allowing the consideration of the care application. This may be explained by the nature of the hospital professional organization that operates as a totalitarian hierarchical system leaving little room for the personal thoughts of individual employees (10, 79). Others have shown that low levels of moral courage in decision-making were associated with defensive medicine (80–82). Defensive medicine could be related to several availability bias components, including legal concerns and apprehension regarding senior staff or family member disapproval. Regarding the presented scenario, HCWs who reported a more substantial effect of items included in the availability bias were more likely to have a more aggressive treatment approach due to their apprehensions regarding the consequences of their choices.

The hypothesis was that the anchoring bias is associated with the treatment approach for persons with AD. In our context, the anchor in the acute care setting focuses on curative care and saving lives, thereby increasing the chances of biasing the thinking in favor of aggressive care (38, 83, 84). This manner of thinking, which frequently happens intuitively and without awareness, occurs from the adaption of the treatment to be accepted and rooted by default. Indeed, when participants presented a concept according to which PC is an accepted norm in their work environment (perceived PC appropriate for persons with AD by colleagues), when they reported comfort with conducting end-of-life conversations, and a lack of accompanying stress and guilt feeling when caring for persons with dementia or when a patient dies, as reflecting an anchor point of thought which is not focused on the obligation to extend and save lives at any cost, this was associated with choosing a more PC approach with less burdensome invasive interventions. Beck et al. (85) found that clinicians in intensive care units involved in critical and/or uncertain situations, such as whether to limit life-sustaining care, have a higher likelihood of choosing passive thinking. This passive approach means preferring non-action to deviation from continuing ongoing care. In this study, the ability to make end-of-life decisions for persons with AD was not associated with the treatment approach. Additionally, contrary to our expectations, we found no association between professional orientation (medical wards’ staff vs. surgical wards’ staff) and the treatment approach. However, this contradicts other studies’ findings, where surgical HCWs delivered more aggressive care than those on medical wards (86, 87). Surgical HCWs are accustomed to operating under a task-oriented, immediate-focused work environment, frequently concentrating on a single organ or system that may limit consideration of long-term consequences (88, 89).

This study found a possible association between the components of knowledge and clinical thinking and the treatment approach. This relationship is essential since the effect of cognitive biases is usually unconscious and has a potentially high risk of shaping perceptions and attitudes. Most attention on cognitive biases relating to medical issues was given to their potential influence on diagnostic errors, with limited research on how they might shape treatment approaches and diminish appropriate ethical reasoning capacity (24). The risk of bias influence and moral reasoning when making decisions for clinically complex ethical situations, such as those involving the course for or against life-sustaining treatment, has been given less attention in research (36, 37). Two reviews on cognitive biases in the context of medical decisions reported that one- to two-thirds of the studies found that risk intolerance or overconfidence resulted in availability, representativeness, and anchoring biases, which may affect the decision process (24, 28). Care decisions are largely based on gut feelings, intuitions, and cognitive biases masquerading as rational and valid arguments affecting the HCW’s judgment (25). A denial of ignorance exists when decisions are based on snap judgments rather than real knowledge. Notably, research evidence has found that, in some cases, an intuitive thinking style is the right approach to making decisions and that obtaining good results depends on matching the thinking pattern with the nature of the task or situation. Intuitive thinking leads to better performance on an intuitive task, and analytical thinking is adapted to an analytical task (90). Since making treatment decisions for complex medical conditions of persons with AD is an inherently analytical task, the HCW should adopt an analytical thinking style. Awareness and recognition of the potential effect of cognitive biases is the first step toward better care decisions and “decision hygiene” (25, 64).

Another important point is the consideration that the effect of the three cognitive biases evaluated may cause the underutilization of potentially beneficent low-burden interventions, such as antimicrobial therapy, in certain circumstances; for example, due to overestimating the severity of dementia in a person with moderate dementia with superimposed delirium secondary to acute medical illness. HCW professionals may withhold potentially beneficial treatment in persons with dementia in a hospice setting or if family members refuse treatment because they are burdened from the daily care or as a reaction to seniors’ opinions and administrative considerations concerning resource allocation. In a study conducted among PC professionals on biases that influence treatment decisions for terminally ill persons, it appears that 9 out of 20 interviewees reported the existence of a bias in favor of PC in a way that may obscure a person or a person’s family preferences (91).

This study had some limitations. First, the design was based on a hypothetical case scenario, and the treatment approach reported may differ from the actual decisions made in real clinical situations. Second, we examined only three cognitive biases, whereas other biases may be involved. Third, the study was conducted at two hospitals in Israel; however, other HCW populations from other countries may respond differently, considering cultural, organizational, and environmental differences. Therefore, this limits the generalizability of the conclusions to other clinical settings. Fourth, the list of medical treatments did not include all possible medical interventions. Other PC aspects associated with non-immediate interventions, such as addressing emotional, spiritual, and/or social and family needs, were excluded from this study.

We would like to emphasize that we do not claim that factors, such as family members’ wishes, organization environment, and cultural and religious considerations, which do not directly result from the person’s medical condition, should be excluded from the comprehensive decision-making related to the treatment approach choice for persons with dementia at the end of life. Indeed, they should be part of a holistic approach to end-of-life debates. However, clinicians should be aware of the potential influence of these factors on the thinking process and should clarify and factor them into the decision process.

In conclusion, when considering acute health problems among persons with AD, it is unsurprising that HCWs’ cognitive biases are associated with the treatment approach. However, the primary sources of these biases appear to be limited knowledge, training, and skills about PC for persons with AD. Therefore, medical education initiatives should be conducted to familiarize HCWs with the thinking processes and common causes of cognitive biases. Future studies should examine the impact of these initiatives and the existence of other cognitive biases in the context of the treatment approach for this population. Clinicians should balance between evidence-based medicine guidelines and patients’ benefits and consider families’ wishes and cultural and religious issues.

## Data availability statement

The raw data supporting the conclusions of this article will be made available by the authors, without undue reservation.

## Ethics statement

The studies involving human participants were reviewed and approved by The ethics committee of Sheba Medical Center and the ethics committee of the Hadassah Medical Center, Affiliated with the Hebrew University of Jerusalem, Faculty of Medicine. The patients/participants provided their written informed consent to participate in this study.

## Author contributions

ME, E-LM, and FD contributed to the conception and design of the study. ME organized the database and wrote the first draft of the manuscript. All authors contributed to the article and approved the submitted version.

## Conflict of interest

The authors declare that the research was conducted in the absence of any commercial or financial relationships that could be construed as a potential conflict of interest.

## Publisher’s note

All claims expressed in this article are solely those of the authors and do not necessarily represent those of their affiliated organizations, or those of the publisher, the editors and the reviewers. Any product that may be evaluated in this article, or claim that may be made by its manufacturer, is not guaranteed or endorsed by the publisher.

## Abbreviations

AD: Advanced dementia
HCW: Health care worker
MSQ: Moral sensitivity questionnaire
PC: Palliative care
PMCS: Professional moral courage scale
