# Supplementary material for: Cognitive biases and moral characteristics of healthcare workers and their treatment approach for persons with advanced dementia in acute care settings
Source: Front Med (Lausanne). 2023 Jun 22;10:1145142. doi: 10.3389/fmed.2023.1145142 (PMC10325688; doi:10.3389/fmed.2023.1145142)
Supplement: Supplementary file 1 [file Data_Sheet_1.pdf]

Supplementary Material

Cognitive biases and moral characteristics of healthcare workers and their treatment approach for persons with advanced dementia in acute care settings

Meira Erel [1†] , Esther-Lee Marcus [2*†], Freda DeKeyser Ganz [1,3]

***Correspondence:** Esther-Lee Marcus: estherlee@herzoghospital.org

Supplemental File 1a

Moral Sensitivity Questionnaire – MSQ (Hebrew version)

נא סמן את התשובה המתאימה ביותר בסולם 1 - 7

1– מסכים 7 – לא מסכים

		1	2	3	4	5	6	7
1	כאיש צוות מקצועי מוטלת עלי האחריות להכיר את המטופל באופן כוללני (הוליסטי)							
2	אין טעם בעבודתי אם לעולם לא אראה שיפור אצל מטופליי							
3	חשוב לי לקבל תגובה חיובית ממטופליי בכל דבר שאני עושה							
4	כאשר עלי לקבל החלטה המנוגדת לרצון המטופל אני בוחר את הטיפול הטוב ביותר לדעתי							
5	אם אאבד את אמון המטופל, אחוש שעבודתי חסרת ערך							
6	כשעלי לקבל החלטות קשות עבור המטופל, חשוב תמיד להיות כן אתו/ה							
7	אני מאמין שטיפול טוב כולל את כיבוד החלטת המטופל							

8	כשהחולה אינו מקבל את מחלתו אני יכול לעשות למענו/ה מעט מאוד							
9	לעיתים קרובות אני עומד בפני מצבים אשר כוללים קונפליקטים הנוגעים להחלטות הטיפול במטופל							
10	אני מאמין בחשיבות קיומם של עקרונות מוצקים ויציבים בנוגע לטיפול בחולים מסוימים							
11	לעיתים קרובות אני נתקל במצבים שבהם קשה לדעת מהי הפעולה האתית הנכונה עבור חולה מסוים							
12	הדבר החשוב ביותר בעבודתי הוא הקשר עם המטופלים							
13	לעיתים קרובות אני נתקל במצבים שבהם קשה לי לאפשר למטופלים לקבל החלטות בעצמם							
14	אני תמיד מבסס את פעולותיי על הידע הרפואי הטוב ביותר הקיים, גם אם המטופל מוחה על כך							
15	אני מאמין שטיפול טוב כרוך לעיתים בקבלת החלטות עבור המטופל							
16	כשאיני בטוח, לרוב אני מסתמך על ידע של בכירים בנוגע לטיפול במטופלים							
17	מעל הכול, התגובות של המטופל הן אלו שמראות לי שקיבלתי את ההחלטה הנכונה							
18	לעיתים קרובות אני חושב על הערכים והנורמות שלי, שעשויים להשפיע על תגובותיי							
19	הניסיון שלי שימושי יותר מידע תיאורטי במצבים שבהם קשה לדעת מה נכון מבחינה אתית							
20	אני מאמין שטיפול טוב כולל את השתתפותו של המטופל, גם בקרב מטופלים עם הפרעות נפשיות חמורות							

21	לעיתים קרובות אני נתקל במצבים קשים שבהם עלי לקבל החלטות ללא שיתוף המטופל							
22	לעיתים ישנה סיבה טובה לאיים על חולה במתן זריקה כאשר הוא מסרב לקבל טיפול תרופתי פומי (אוראלי)							
23	אני מוצא שקשה לתת טיפול טוב נגד רצונו של מטופל							
24	במצבים שבהם קשה לדעת מה נכון, אני מתייעץ עם עמיתיי בנוגע לדרך הפעולה							
25	כאשר אני צריך לקבל החלטה קשה עבור מטופל אני מסתמך בעיקר על האינטואיציה שלי							
26	כאיש צוות רפואי, עלי לדעת תמיד כיצד לפנות בכבוד לכל אחד ממטופליי							
27	בפעולותיי ישנה משמעות גם כאשר אינני יכול לפתח אצל המטופל מודעות בנוגע למחלתו							
