# Supplementary material for: Cognitive biases and moral characteristics of healthcare workers and their treatment approach for persons with advanced dementia in acute care settings
Source: Front Med (Lausanne). 2023 Jun 22;10:1145142. doi: 10.3389/fmed.2023.1145142 (PMC10325688; doi:10.3389/fmed.2023.1145142)
Supplement: Supplementary file 2 [file Data_Sheet_2.pdf]

Supplementary Material

Cognitive biases and moral characteristics of healthcare workers and their treatment approach for persons with advanced dementia in acute care settings

Meira Erel [1†] , Esther-Lee Marcus [2*†], Freda Dekeyser Ganz [1,3]

***Correspondence:** Esther-Lee Marcus: estherlee@herzoghospital.org

Supplemental File 1b

Professional Moral Courage Scale -PMCS (Hebrew version)

נא סמן את התשובה המתאימה ביותר בסולם 1 - 7

1 – אף פעם   7 - תמיד

		1	2	3	4	5	6	7
1	אני אדם שפועל ללא הרף למען עשיית הדבר הנכון בעבודה.							
2	כשאני עובד אני משתמש באופן קבוע באמצעים נוספים על מנת להבטיח שפעולותיי יפחיתו נזק לאחרים.							
3	עמיתיי לעבודה מתארים אותי כמי שעושה תמיד כל שניתן להשגת ביצוע אתי, שעושה כל מאמץ לעבוד באופן מכובד.							
4	אני אדם המשתמש בעקרונות ובהנחיות של הארגון כאשר אני מקבל החלטות אתיות בעבודה.							
5	בכל מקרה, אני משתדל להביא בחשבון הן את ערכי הארגון שבו אני עובד והן את ערכיי האישיים בזמן קבלת החלטות.							

6	לעיתים קרובות בעת קבלת החלטות אני בוחן כיצד מיושמים בפעולה הסופית תפקידיי בארגון, דעתו של הממונה עלי והרקע שבו חונכתי							
7	כאשר אני עומד אל מול אתגר אתי, אני פועל בדרך מוסרית, בלי להתחשב בהשפעה השלילית שעשויה לנבוע מכך על הדרך שבה רואים אותי האחרים							
8	אני דבק בדרכי המוסרית, גם בעת קיומם של לחצים חברתיים המנוגדים לה.							
9	אני פועל באופן מוסרי גם במקרים שבהם פעולותיי עשויות להעמיד אותי בעמדה לא נוחה אל מול הממונים עלי.							
10	הקולגות שלי לעבודה אומרים שכשאני מבצע את עבודתי אני עושה יותר ממה שהתקנות דורשות. אני עושה כל שביכולתי למען הבטחת ההיבט המוסרי של פעולותיי.							
11	בביצוע מטלות עבודה יומיות, אני מוודא ציות לכללים, אך גם מנסה להבין את כוונתם, על מנת להבטיח שייעשו כפי שנדרש							
12	חשוב לי לפעול מעבר לדרישות הכתובות בחוק ולהשיג רמה אתית גבוהה בפעולותיי							
13	בקבלת החלטות בעבודה חשוב לי להשתמש בשיקול דעת							
14	אני בוחן את מניעיי בביצוע משימה כדי להבטיח ביסוס מוסרי לעשייתי							
15	אני פועל בדרך מוסרית כי זה הדבר הנכון לעשות							
