# Supplementary material for: Cognitive biases and moral characteristics of healthcare workers and their treatment approach for persons with advanced dementia in acute care settings
Source: Front Med (Lausanne). 2023 Jun 22;10:1145142. doi: 10.3389/fmed.2023.1145142 (PMC10325688; doi:10.3389/fmed.2023.1145142)
Supplement: Supplementary file 3 [file Data_Sheet_3.pdf]

frontiers

Supplementary Material

Cognitive biases and moral characteristics of healthcare workers and their treatment approach for persons with advanced dementia in acute care settings

Meira Erel [1†] , Esther-Lee Marcus [2*†], Freda DeKeyser Ganz [1,3]

***Correspondence:** Esther-Lee Marcus: estherlee@herzoghospital.org

Supplemental File 2a

Questionnaire 4- Case Scenario

You are in charge of an 85-year-old male patient diagnosed with dementia 5 years ago. The patient is a nursing home resident, who cannot communicate verbally, needs full assistance with mobility and basic functioning, and experiences urinary and bowel incontinence. He also has several pressure sores and is fed via a percutaneous endoscopic gastrostomy.

Subsequently, the patient was admitted to the emergency room because of pneumonia and shortness of breath. He is currently in respiratory failure and is oliguric. Furthermore, he is currently in severe respiratory distress, and his oxygen saturation is 85% on a 40% oxygen face mask.

From the interventions/treatment alternatives listed below, please choose the ones you would select in the abovementioned case:

1. Intubation and mechanical ventilation*
2. Intravenous fluid infusion**
3. Antimicrobial therapy**
4. Laboratory tests**
5. Analgesia***
6. Sedation***

*Scored 3 in the Treatment Approach Score

**Scored 2 in the Treatment Approach Score

*** Score (-1) if selected and +1 if not selected
