# Supplementary material for: Cognitive biases and moral characteristics of healthcare workers and their treatment approach for persons with advanced dementia in acute care settings
Source: Front Med (Lausanne). 2023 Jun 22;10:1145142. doi: 10.3389/fmed.2023.1145142 (PMC10325688; doi:10.3389/fmed.2023.1145142)
Supplement: Supplementary file 4 [file Data_Sheet_4.pdf]

frontiers

Supplementary Material

Cognitive biases and moral characteristics of healthcare workers and their treatment approach for persons with advanced dementia in acute care settings

Meira Erel [1†] **, Esther-Lee Marcus** [2*†]**, Freda DeKeyser Ganz** [1,3]

***Correspondence:** Esther-Lee Marcus: estherlee@herzoghospital.org

Supplemental File 2b

Case scenario – Hebrew version

הינך מופקד/ת על הטיפול במטופל בן 85, אשר מאובחן עם דמנציה מזה חמש שנים. המטופל הוא דייר בית אבות סיעודי, אינו יכול לתקשר מילולית, מאופיין בתלות מלאה בניידות וכלל הפעולות הבסיסיות היום-יומיות ואי-שליטה על סוגרים. המטופל סובל מכמה פצעי לחץ. המטופל מוזן דרך פיום קיבה (PEG) .

המטופל התקבל למחלקה לרפואה דחופה בשל דלקת ריאות וקוצר נשימה. הוא כעת במצב של כשל נשימתי וכן ירידה בתפוקת השתן (אוליגוריה). בבדיקה המטופל במצוקה נשימתית ורמת רוויון החמצן בדם במתן חמצן 40%, במסיכה – 85% .

מבין חלופות ההתערבות/הטיפול הבאות בחר/י את החלופות המתאימות למטופל שהוצג :

1. החדרת טובוס והנשמה מלאכותית*
2. מתן נוזלים תוך ורידי**
3. טיפול אנטיביוטי**
4. בדיקות מעבדה**
5. אנלגטיקה***
6. תרופות הרגעה***

*ניקוד 3 בסולם הגישה הטיפולית

**ניקד 2 בסולם הגישה הטיפולית

***ניקוד (-1) אם נבחר; 1 אם לא נבחר בסולם הגישה הטיפולית
